# Antimicrobial Peptide Reduces Cytotoxicity and Inflammation in Canine Epidermal Keratinocyte Progenitor Cells Induced by *Pseudomonas aeruginosa* Infection

**DOI:** 10.3390/vetsci11060235

**Published:** 2024-05-23

**Authors:** Jae-Eun Hyun, Cheol-Yong Hwang

**Affiliations:** 1 Department of Veterinary Internal Medicine, College of Veterinary Medicine, Konkuk University, Seoul 05029, Republic of Korea; 2 Laboratory of Veterinary Dermatology, Research Institute for Veterinary Science, College of Veterinary Medicine, Seoul National University, Seoul 08826, Republic of Korea

**Keywords:** antimicrobial peptides, pseudomonas aeruginosa, lipopolysaccharides, antibacterial activity, antibiofilm effect, dogs, keratinocytes

## Abstract

**Simple Summary:**

*Pseudomonas aeruginosa* is a representative Gram-negative bacterial species that causes chronic deep infections in the skin and ears of dogs. Increasing *P. aeruginosa* antibiotic resistance in human and veterinary medicine requires the identification of new antibacterial substances. In this study, we demonstrated the antibiotic and antibiofilm activities of synthetic canine antimicrobial peptides (AMPs) against *P. aeruginosa*. In addition, it was confirmed that AMPs significantly reduced the cell toxicity induced by *P. aeruginosa* and reduced the *P. aeruginosa* lipopolysaccharide (LPS)-induced inflammation in canine keratinocytes. These findings suggest the potential of AMPs as a new antibacterial agent for the *P. aeruginosa* infection of canine skin.

**Abstract:**

The direct effects and antimicrobial activity of synthetic antimicrobial peptides (AMPs) obtained from dogs, including cBD, cBD103, and cCath, against *P. aeruginosa* wild-type strain PAO1 and canine keratinocytes were analyzed. Antibacterial effects on planktonic bacteria were assessed by determining the minimum bactericidal concentrations (MBCs) of AMPs and by a time-kill assay. Antibiofilm effects were assessed using the microtiter plate assay. We also evaluated the effects of AMPs on cell cytotoxicity and host immune response induced by stimulating canine epidermal keratinocyte progenitor (CPEK) cells with PAO1 and its LPS. cBD, cBD103, and cCath all exhibited dose-dependent antimicrobial and antibiofilm effects. In particular, 25 μg/mL cBD103 showed rapid bactericidal activity within 60 min and inhibited biofilm formation. In addition, pretreatment with cBD103 (25 µg/mL) and cCath (50 µg/mL) 1 h before stimulation significantly reduced the cytotoxicity of the CPEK cells by PAO1 and LPS-induced IL-6 and TNF-a expressions. cBD had little effect on the response to PAO1 and LPS in the cells. These results indicate the therapeutic potential of AMPs in *P. aeruginosa* skin infections. However, further studies on the mechanism of action of AMPs in keratinocytes and clinical trials are needed.

## 1. Introduction

Antimicrobial peptides (AMPs) are small endogenous peptides produced by cells in various animal tissues, and numerous types have been reported [1]. In the skin, host defense molecules, such as AMPs, synthesized by resident skin cells, such as keratinocytes, and recruited inflammatory cells participate in the innate cutaneous immune defense [2,3]. Cationic AMPs, in particular, contain a high concentration of positively charged amino acids that exert antibacterial action [4]. Positively charged AMPs initiate antibacterial activity by engaging negatively charged bacterial structures, such as lipopolysaccharides (LPSs), phospholipids, and teichoic acid. Cutaneous barrier defects result in abnormal AMP secretion, consequently rendering skin vulnerable to infection [3,5]. Because of their complex structures and different modes of action against target cells, AMPs make it extremely difficult for pathogens to gain resistance [1]. The versatility of AMPs highlights their potency as alternatives to antibacterial drugs [6]. Several studies were conducted on the effects of AMPs against problematic antibiotic-resistant bacteria [6,7,8].

*Pseudomonas aeruginosa* is one of the most problematic bacterial agents that infect the human respiratory system and cause nosocomial infections, especially in immunosuppressed patients [9]. Several studies investigated the mode of action of *P. aeruginosa* in the human respiratory tract, including the effects of various virulence factors on epidermal cells [10,11]. In *P. aeruginosa* infection, the bacteria not only directly affect the epidermal cells but also induce an inflammatory response through the explosive formation of inflammatory intermediaries through cell signaling pathways. However, the inflammatory response caused by host immunity against *P. aeruginosa* is limited to removing the bacteria to overcome the infection [7]. In addition, the formation of a pseudomonal biofilm facilitates infection through several mechanisms, including protecting the bacteria within the biofilm and the acquisition of antibiotic resistance [12,13]. *P. aeruginosa* is a major bacterial causative agent of deep infections of the skin and ears in dogs [14,15]. An increase in *P. aeruginosa* antibiotic resistance and the emergence of multidrug-resistant *P. aeruginosa* have been reported in dogs [16]. Due to resistance to existing antibiotics, the development of new antibacterial treatments is an emerging necessity.

This study evaluated the antibacterial activity against *P. aeruginosa* of AMPs synthesized from the sequence of beta-defensin and cathelicidin in dogs. We also analyzed the direct antibacterial properties of synthetic peptides against *P. aeruginosa* and their inhibitory effects on pseudomonal biofilm formation. Furthermore, we investigated the effects of AMPs on cellular and inflammatory changes in canine keratinocytes infected with *P. aeruginosa* and its bacterial components.

## 2. Materials and Methods

### 2.1. Bacterial Strains and Reagents

The *P. aeruginosa* wild-type strain PAO1 used in this study was kindly provided by Professor Dr. Sang Sun Yoon of the Yonsei University College of Medicine, Seoul, Republic of Korea. For the in vitro bactericidal and antibiofilm assay, *P. aeruginosa* was cultured overnight in Luria Bertani (LB; Becton Dickson, Sparks, MD, USA) broth at 37 °C with shaking until the stationary phase was reached. Bacterial suspensions for infecting cells were prepared as described previously [7]. The concentration of the overnight culture was adjusted to an optical density (OD) of 0.1 (1 × 10^9^ colony forming units (CFUs)/mL) at 600 nm in a Beckman spectrophotometer (Beckman Coulter, Brea, CA, USA), and the supernatant was removed after centrifugation at 450 × *g* for 10 min. After washing three times with sterile phosphate-buffered saline (PBS), the pellet was resuspended in antibiotic-free cell culture medium. The resuspension was diluted to 10^6^–10^8^ CFU/mL immediately before the cell infection.

Preparation of LPS isolated from *P. aeruginosa* (L9143, Sigma-Aldrich, St. Louis, MO, USA) was performed as described previously with some modifications [7]. The LPS was dissolved at a concentration of 1 mg/mL and stored at 4 °C until use. Endotoxin-free water (InvivoGen, San Diego, CA, USA) was used for the initial dissolution, and antibiotic-free culture medium was used for further dilution for cell experiments.

### 2.2. Peptide Synthesis

All peptides were synthesized by solid-phase F-moc chemistry at Lugen Sci Co., Ltd. (Bucheon, Republic of Korea). Each synthetic peptide was subsequently purified to greater than 95% on a reverse-phase high-performance liquid chromatography system. Then, each peptide mass was determined by mass spectroscopy. The sequences of the peptides were derived from canine beta-defensin and cathelicidin as previously described [8] and are presented in Table 1. The peptides were stored as desiccated powders before use. For the in vitro bactericidal experiments, the desiccated powders were suspended in 10 mm of 0.01% acetic acid (Sigma-Aldrich) to a final concentration of 1 mg/mL and then further diluted with 10 mM sodium phosphate buffer (SPB, pH 7.4). However, for the cell experiments, desiccated powders were suspended in endotoxin-free water (InvivoGen) immediately before use [6]. All peptide dilutions were stored at −20 °C in 100 µL aliquots until further use.

### 2.3. Evaluation of Direct Antibacterial and Antibiofilm Activities of Synthetic Peptides against P. aeruginosa PAO1

#### 2.3.1. Effects of Antimicrobial Peptides on Planktonic *P. aeruginosa* PAO1

To determine the antimicrobial activity of AMPs against *P. aeruginosa*, the minimum bactericidal concentration (MBC) was determined, and the time-kill assay was performed as described previously with some modifications [17]. Briefly, the cultures were grown overnight, centrifuged at 5000 rpm for 5 min, washed once with PBS, and the pellet obtained was suspended in LB medium. The OD of the bacterial suspension was adjusted to 0.1 at 600 nm. Each well of 96-well round-bottom plates (SPL, Seoul, Republic of Korea) was inoculated at 5 × 10^5^ CFU/mL. Peptides were added by serial dilution to the bacterial suspension at concentrations from 6.25 to 100 µg/mL. After incubation at 37 °C for 2 h, 20 µL of each culture medium was subcultured in tryptic soy agar with 5% sheep blood (Hangang, Gunpo, Republic of Korea). The agar plates were incubated aerobically at 37 °C for 24 h, and the number of colonies was counted. The MBC was defined as the lowest concentration at which 99.9% of the test bacteria were killed.

To evaluate the time-killing effect of AMPs, each one was inoculated at its MBC into 5 × 10^5^ CFU/mL of PAO1 and incubated for a specified time to evaluate the change in CFUs as described above. The CFUs were measured every 10 min for 60 min, and every 30 min for 180 min thereafter. The peptide-free SPB solution described earlier was used as a negative control in both assays. All experiments were performed independently in triplicate.

#### 2.3.2. Biofilm Formation Assay

The antibiofilm effects of AMPs were evaluated using the 96-well microtiter plate assay as previously described with some modifications [18]. Briefly, PAO1 was incubated in LB medium overnight. When the culture reached a stationary phase, it was diluted 1:100 in fresh LB medium. The culture (100 μL) was dispensed into four replicate wells in 96-well microtiter plates (SPL), and AMPs were added at serially diluted concentrations (range, 6.25–50 µg/mL). After incubation for 22 h at 37 °C, planktonic bacteria were removed. The biofilm was stained with crystal violet, and the absorbance was measured at 595 nm using a microplate absorbance reader (Bio-Rad, Munich, Germany). All experiments were performed independently in triplicate.

### 2.4. Effects of AMPs of Keratinocytes

#### 2.4.1. Cell Culture

Canine epidermal keratinocyte progenitor (CPEK) cells were purchased from CELLnTEC Advanced Cells Systems (Bern, Switzerland). The cells were cultured in keratinocyte culture medium (CnT-09, CELLnTEC) according to the manufacturer’s instructions. CPEK cells were plated into 12- or 24-well tissue culture plates (SPL) at a density of approximately 1 × 10^5^ cells/cm^2^ and incubated at 37 °C in a 5% CO_2_ humidified atmosphere until the cells reached 80–90% confluency. Cells between the fifth and seventh passages were used.

#### 2.4.2. Cytotoxicity of AMPs to CPEK Cells

The cytotoxicity of AMPs to CPEK cells was measured using the EZ-Cytox cell viability kit (Daeil Laboratories, Seoul, Republic of Korea) based on the water-soluble tetrazolium salt (WST) assay according to the manufacturer’s instructions. CPEK cells were seeded in 96-well plates at a density of 1 × 10^5^ cells/cm^2^ and incubated for 24 h at 37 °C under 5% CO_2_. The cells were treated with various concentrations of AMPs and incubated for 24 h at 37 °C under 5% CO_2_. Subsequently, 10 µL of EZ-Cytox reagent was added to each well. After further incubation for 4 h at 37 °C, the absorbance was measured at 450 nm using a microplate reader (Bio-Rad). The culture medium and 2% (v/v) Triton X-100 (Sigma-Aldrich, St. Louis, MO, USA) were used as negative and positive controls, respectively.

#### 2.4.3. Effects of AMPs on the Cytotoxicity of *P. aeruginosa*

CPEK cells were seeded in 96-well culture plates at a density of 1.3 × 10^4^ cells/well and incubated for 24 h. To determine the best multiplicity of infection (MOI) and infection timepoint of PAO1 in CPEK cells, CPEK cells were incubated with bacterial suspensions at MOIs of 0.1, 1, 10, and 100. After incubation (2, 4, or 6 h), the cytotoxicity of *P. aeruginosa* to CPEK cells was evaluated by measuring the release of lactate dehydrogenase (LDH) using the EZ-LDH cell cytotoxicity assay kit (Daeil Laboratories) according to the manufacturer’s instructions. The cell-free supernatants were collected and centrifuged at 1000 rpm for 5 min. Subsequently, a 10 µL aliquot of each supernatant was reacted with 100 µL of the reaction mixture for 30 min in the dark. The cell culture medium was used as a negative control (0% toxicity), and 2% *v*/*v* Triton X-100 was used as a positive control (100% toxicity). The absorbance of the reaction was measured at 450 nm using a microplate reader (Bio-Rad). The effect of AMPs on the cytotoxicity of PAO1 was evaluated. The CPEK cells were seeded in 96-well culture plates at a density of 1.3 × 10^4^ cells/well and incubated for 24 h. One hour before the cells were infected with *P. aeruginosa*, cells were pretreated with cBD (50 µg/mL), cBD103 (25 µg/mL), or cCath (50 µg/mL). Then, the cells were co-cultured with PAO1 at a MOI of 1 for 4 h. After 4 h incubation, the cell-free supernatants were collected, and the LDH assay was performed as above. All assays were performed in three independent experiments.

#### 2.4.4. Determination of Cytokine Expression

CPEK cells were seeded in 96-well plates at a density of 1.0 × 10^6^ cells/well and incubated at 37 °C in 5% CO_2_. After 24 h incubation, the cell culture medium was replaced with fresh medium, and AMPs were added at various concentrations. After 1 h, *P. aeruginosa* LPS (1 µg/mL) was added to the cells and incubated for a designated time (6 and 24 h). The supernatants were removed and stored at −20 °C until used in an enzyme-linked immunosorbent assay (ELISA). Cytokines secreted by CPEK cells in supernatants were quantified using ELISA kits (R&D Systems, Minneapolis, MN, USA) according to the manufacturer’s instruction.

### 2.5. Statistical Analysis

Data were statistically analyzed using the software program IBM SPSS Statistics version 23 for Windows (IBM Corp., Armonk, NY, USA). GraphPad Prism version 8 (GraphPad Software, Inc., La Jolla, CA, USA) was used to perform one- or two-way analysis of variance. Post hoc analysis was performed using Tukey’s and Dunnett’s multiple comparisons tests. Data are expressed as mean ± standard deviation. A *p*-value < 0.05 was considered statistically significant.

## 3. Results

### 3.1. AMPs Exerted Bactericidal Activity on Planktonic P. aeruginosa

**Figure 1:**
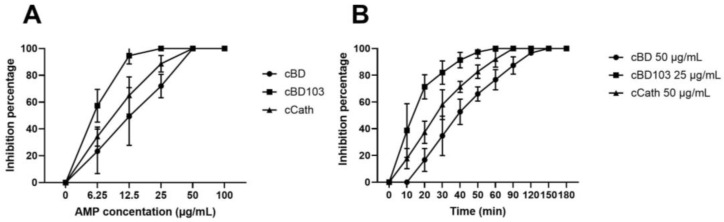
(**A**) Antimicrobial activity of cBD, cBD103, and cCath on PAO1. (**B**) Time-kill assay against Pseudomonas aeruginosa.

The treatment of *P. aeruginosa* cultures with AMPs for 24 h showed that all AMPs exerted dose-dependent inhibition of the bacterial growth (Figure 1A). Differences were found in the concentrations of *P. aeruginosa* inhibited by the AMPs. The bacterial growth was completely inhibited at 25 µg/mL of cBD103, whereas, for cBD and cCath, the bacterial growth was inhibited at 50 µg/mL. The bactericidal kinetics of AMPs were evaluated using the time-kill assay at the concentration of complete inhibition (Figure 1B). cBD103 completely inhibited the bacterial growth at 25 μg/mL within 60 min. In contrast, 50 μg/mL cCath and 50 μg/mL CBD took 90 min and 150 min to inhibit the bacterial growth, respectively.

### 3.2. AMPs Suppressed Pseudomonal Biofilm Formation

**Figure 2:**
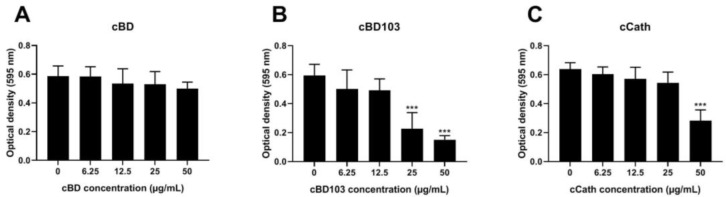
Inhibitory activity against pseudomonal biofilm formation of (**A**) cBD, (**B**) cBD103, and (**C**) cCath. Biofilm formation was evaluated by crystal violet staining. The absorbance was measured at 595 nm using a microplate absorbance reader. All experiments were performed independently in triplicate and analyzed using one-way analysis of variance with Tukey’s multiple comparisons. Results are expressed as mean ± standard deviation. *** *p* < 0.001.

All AMPs dose-dependently reduced the biofilm formation by PAO1 (Figure 2). However, no significant reduction in biofilm formation was observed at any cBD concentration used in this experiment (Figure 2A). In contrast, cBD103 and cCath significantly reduced the biofilm viability at concentrations of 25 μg/mL and 50 μg/mL, respectively (Figure 2B,C).

### 3.3. AMPs Alleviated the Cytotoxicity of Canine Keratinocytes Induced by PAO1

**Figure 3:**
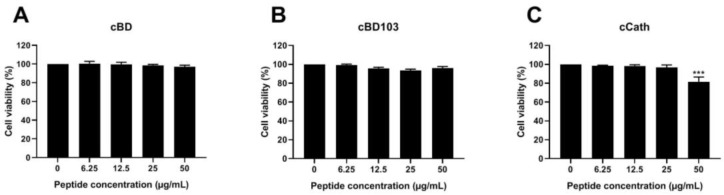
Effects of (**A**) cBD, (**B**) cBD103, and (**C**) cCath on the viability of CPEK cells. Cell viability and proliferation were evaluated using an EZ-Cytox cell viability kit. Values are expressed as the mean ± standard deviation and were analyzed using one-way analysis of variance and Tukey’s multiple comparisons test in three independent experiments. *** *p* < 0.001.

**Figure 4:**
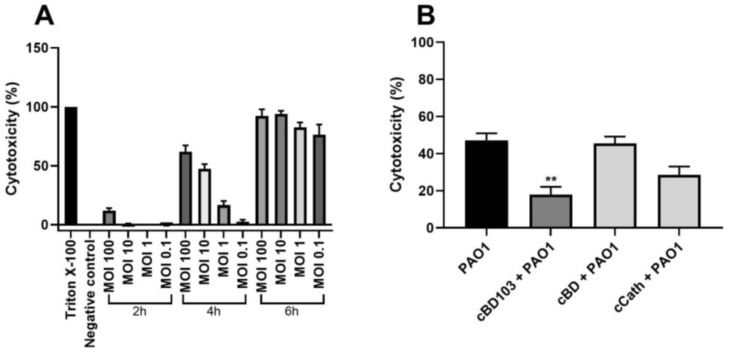
*Pseudomonas aeruginosa*-induced cytotoxicity of CPEK cells. (**A**) Infectivity of *P. aeruginosa* in CPEK cells was established by inoculating PAO1 at multiples of infection of 0.1, 1, 10, or 100 for a designated time (2, 4, or 6 h). (**B**) The effect of antimicrobial peptides on *P. aeruginosa*-induced cytotoxicity on CPEK cells. Cell cytotoxicity was assessed using an EZ-LDH cell cytotoxicity assay kit. Results are expressed as mean ± standard deviation and were analyzed using one-way analysis of variance and Tukey’s multiple comparisons test in three independent experiments. ** *p* < 0.01.

After the CPEK cells were treated with AMPs at various concentrations, the cell viability was verified by a WST assay. The AMPs themselves exhibited little to no cytotoxic effects on the CPEK cells (Figure 3). Interestingly, however, after the treatment with cCath at 50 μg/mL, the CPEK cell viability decreased to 80% (Figure 3C). PAO1 was inoculated into CPEK cells at an MOI of 1 for 4 h based on the results that determined the cell infectivity of PAO1 in the CPEK cells (Figure 4A). The cytotoxicity of keratinocytes after bacterial stimulation was further confirmed using the LDH assay. cBD103 at a 25 μg/mL concentration significantly reduced the cell toxicity induced by PAO1, whereas 50 μg/mL cCath also decreased the cytotoxicity but not significantly (Figure 4B). In contrast, cBD had little effect on the cytotoxicity.

### 3.4. AMPs Mitigated P. aeruginosa LPS-Induced Inflammation in Canine Keratinocytes

**Figure 5:**
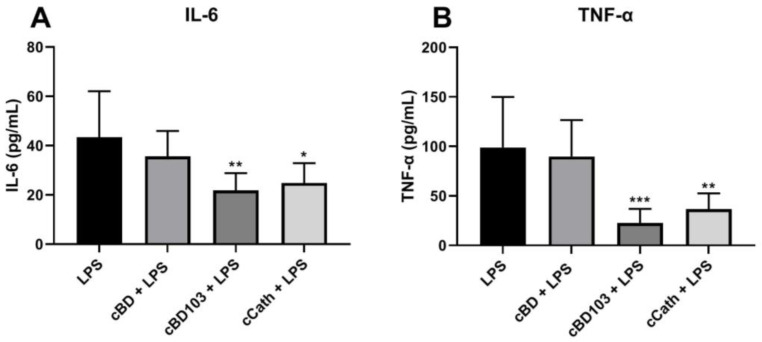
Cytokine release following stimulation with lipopolysaccharide (LPS) derived from *Pseudomonas aeruginosa*. The releases of (**A**) IL-6 and (**B**) TNF-α were quantified in the cell supernatants using an enzyme-linked immunosorbent assay. Antimicrobial peptides were pretreated 1 h before the LPS stimulation. Data are expressed as the mean ± standard deviation. Data were analyzed in three independent experiments conducted in triplicate using one-way analysis of variance with Tukey’s multiple comparisons. * *p* < 0.05; ** *p* < 0.01; *** *p* < 0.001.

The expression of proinflammatory cytokines was analyzed by ELISA after 6 or 24 h of stimulation with 1 µg/mL *P. aeruginosa* LPS. LPS significantly increased IL-6 and TNF-α expression in keratinocytes, with no difference in the degree of cytokine expression between the two time points (Figure 5). After the cells were pretreated with AMPs 1 h before LPS stimulation, the changes in the expression of proinflammatory cytokines were analyzed. The addition of cBD103 and cCath significantly reduced both IL-6 and TNF-α expressions. However, cBD had no significant effect on the cytokine expression.

## 4. Discussion

Several studies investigated the potential application of AMPs as therapeutic agents and their effects on the immune response of the hosts [1,7]. Although the excellent antimicrobial effects of AMPs for pathogens, such as bacteria, viruses, and fungi, were demonstrated [3,19,20,21], studies on AMPs in veterinary medicine are limited. In the present study, we investigated the effects of AMPs on *P. aeruginosa* and on canine keratinocytes infected with *P. aeruginosa*. cBD, cBD103, and cCath, which were the synthetic cationic peptides used in this experiment, were derived from canine beta-defensin sequences and canine cathelicidin, which are produced and secreted in the skin of dogs [8,22]. All three peptides showed bactericidal effects against *P. aeruginosa*, as previously reported [8] (Figure 1A). Several studies showed that the antimicrobial effects of synthetic peptides vary with the type of peptide and strains and phenotype of bacteria used in the experiments [17,23,24]. The time-kill assay in this study showed that the onset times of the bactericidal effects of cBD and cCath were slightly slower than those reported previously [8]. However, cBD showed excellent antibacterial activity against *P. aeruginosa* within 1 h (Figure 1B). It has been reported that the antibacterial activity of AMPs is mainly caused by selective disruption of the cell membrane of the pathogens and by pore formation [17,25]. However, further research is required to address the mechanism of action of canine AMPs on pathogens. Furthermore, it has been reported that culture media can influence the chemical stability and minimum inhibitory concentration of compounds [26]. Therefore, additional research is necessary to verify the stability and antibacterial efficacy of canine AMPs in various culture media.

A pseudomonal biofilm not only acts as a barrier against antimicrobial agents but also promotes the acquisition of antibiotic resistance genetically [27]. The characteristic biofilm formation of *P. aeruginosa* is particularly problematic because it causes a treatment-refractory alert detection in dogs [28,29]. Similar to their antibacterial effect on planktonic *P. aeruginosa*, cBD103 and cCath inhibited biofilm formation, with cBD103 especially demonstrating excellent inhibitory activity at lower doses (12.25 µg/mL) (Figure 2). In contrast, cBD exhibited poor biofilm suppression, even at higher doses (Figure 2A). Further studies are needed to elucidate the mechanism of suppressing pseudomonal biofilm formation by AMPs.

Various synthetic AMPs have earned significant research attention for the past decade or so. Low cytotoxicity and high permeability to tissues and suitability for a wide range of microbial diseases are the key characteristics to the therapeutic efficacy of AMPs [30]. This study also demonstrated the low cytotoxicity of AMPs to keratinocytes; however, a slight decrease in cell viability was observed at higher concentrations of cCath (Figure 3C). A study described the synergistic cytotoxic effect of human cathelicidin LL-37 when combined with a *P. aeruginosa* strain 103 [6]. The synergistic cytotoxic effect of cCath and PAO1 was not demonstrated in this study. Although cCath at 50 µg/mL decreased the viability of the CPEK cells, it reduced the cytotoxicity of the CPEK cells inoculated with PAO1. However, further investigation into the cytotoxic potential of high concentrations of cCath on canine keratinocytes over an extended period is necessary before clinical application. The cytotoxicity of the CPEK cells was significantly decreased with the pretreatment of keratinocytes with cBD103 1 h before *P. aeruginosa* exposure (Figure 4). cBD itself did not exhibit cytotoxicity to the CPEK cells or affect the cytotoxicity of keratinocytes induced by PAO1.

We demonstrated that LPS isolated from *P. aeruginosa* increased the expressions of IL-6 and TNF-α, which are major proinflammatory cytokines that activate and coordinate the skin immune response against bacteria, in canine keratinocytes. However, the cytokine levels significantly decreased with AMP pretreatment at 1 h before the LPS was added. cBD103 and cCath reduced the inflammatory response in the CPEK cells, with similarities between the 6 and 24 h time points. Since the intracellular uptake of LPS occurs within 1 h [31], AMPs may have influenced the signaling pathways that induce the inflammatory reactions of keratinocytes rather than directly exerting activity against LPS, as was demonstrated in mouse macrophage cells and human bronchial epithelial cells [6]. As demonstrated in the antibacterial effects or cytotoxicity assay, cBD had little effect on the cytokine secretion.

The *P. aeruginosa* wild-type PAO1 strain was used in this study. Most of the pathogens isolated from patients with pulmonary cystic fibrosis were wild-type *P. aeruginosa*; however, in chronic infection, the bacteria showed diversification, such as conversion to a mucoid phenotype [17,32], which can induce a more vigorous inflammatory reaction in cells [7]. Although bacteria are very unlikely to acquire resistance to AMPs [1], a previous study reported that mucoid *P. aeruginosa* shielded nonmucoid variants and enhanced the resistance to AMPs [33]. Although AMPs exert superior antimicrobial effects against various pathogens, including multidrug-resistant bacteria, the effects of AMPs can vary slightly depending on the bacterial phenotype [17,23]. In addition, there are several phenotypic differences between the PAO1 strain and clinical isolates from humans and dogs [34]. Further studies are still needed to fully understand the efficacy of AMPs against clinical isolates from canine skin, which hold various virulence factors.

## 5. Conclusions

We demonstrated the direct effects of synthetic AMPs derived from canine AMP sequences on the *P. aeruginosa* wild-type strain PAO1 and elucidated the effect of PAO1 infection on cells. cBD, cBD103, and cCath all demonstrated antibiotic and antibiofilm activities comparable with those reported for human AMPs, with cBD103 exhibiting particularly rapid action at low doses. cBD103 was also noncytotoxic in itself but significantly reduced the cell toxicity of the CPEK cells inoculated with *P. aeruginosa*. cCath exerted some cytotoxicity at relatively higher concentrations (50 µg/mL) but also reduced the cytotoxicity of the CPEK cells infected with *P. aeruginosa*. CBD showed weak or no cytotoxicity effects on the keratinocytes induced by *P. aeruginosa*, as well as low antibiofilm suppression. These results demonstrate the therapeutic potential of AMPs with little to no cytotoxic effects on canine keratinocytes in the treatment of cutaneous *P. aeruginosa* infection in dogs. However, further studies of the chemical stability of AMPs and the optimal conditions for maximal effects, as well clinical trials in animal models, are required to confirm the current findings.

## Figures and Tables

**Table 1:** Peptide sequences used in this study.

Peptide Name	Sequence	Molecular Weight *	NCBI Reference Sequence
cBD	KCWNLRGSCREKCIKNEKLYIFCTSGKLCCLKPK	3994.92	NM_001313788.1 (202–303, 102 bp)
cBD103	GIINTLQRYYCRIRSGRCALLSCLPKEEQIGRCSSTGRKCCRRKK	5206.23	NM_001129980.1 (180–314, 135 bp)
cCath	RLKELITTGGQKIGEKIRRIGQRIKDFFKNLQPREEKS	4512.29	NM_001003359.1 (443–556, 114 bp)

* Molecular weights were determined by mass spectroscopy.

## Data Availability

The data presented in this study are available upon request from the corresponding author.
